# Facteurs obstétricaux, infectieux et traumatiques associés à l’épilepsie dans la zone rurale de Bangoua (Ouest, Cameroun)

**DOI:** 10.11604/pamj.2014.19.389.4090

**Published:** 2014-12-18

**Authors:** Kuate-Tegueu Callixte, Tsinkou Huguette Charlie, Kouemeni Lysette, Nguefack-Tsague Georges, Kaptue Lazare, Takougang Innocent

**Affiliations:** 1 Faculté de Médecine et des Sciences Biomédicales, Université de Yaoundé I, Cameroun; 2 Institut Supérieur des Sciences de la Santé, Université des Montagnes, Bangangté, Cameroun

**Keywords:** Epilepsie, Cameroun, facteur de risque, étiologie, Bangoua, zone rurale, Epilepsye, Cameroon, risk factor, etiologie, Bangoua, rural area

## Abstract

**Introduction:**

Les étiologies des épilepsies sont très variées et résultent de la conjonction de facteurs génétiques, périnataux, les anomalies du développement cortical, et des lésions acquises du cortex cérébral. La présente étude a été conçue pour déterminer les facteurs obstétricaux, infectieux et traumatiques pouvant expliquer la prévalence élevée de l’épilepsie à Bangoua.

**Méthodes:**

La présente étude cas-témoins a été réalisée dans la localité de Bangoua, département du Ndé, région de l'Ouest du Cameroun. Les patients épileptiques consentants et les témoins non épileptiques appariés selon l’âge et le sexe ont été recrutés du 4 août au 20 octobre 2008. Le diagnostic d’épilepsie était retenu lorsqu'un patient avait rapporté au moins deux crises d’épilepsie non provoquées au cours des deux dernières années.

**Résultats:**

L’âge des patients variait de 6 à 65 ans avec une moyenne de 26,7 ± 10,6 ans. Le sexe masculin prédominait chez les patients épileptiques (54,3%). Plus de la moitié (57,1%) des patients épileptiques avaient des antécédents familiaux d’épilepsie et les atteintes infectieuses du système nerveux central étaient deux fois plus fréquentes (p = 0,005) chez les participants épileptiques (38,6%) que chez les témoins (17,4%).

**Conclusion:**

La présente étude a relevé que dans la localité de Bangoua, un antécédent familial d’épilepsie, le paludisme, une infection urinaire ou une éclampsie pendant la grossesse, un antécédent d'encéphalite sont des facteurs associés à l’épilepsie.

## Introduction

L’épilepsie est une affection neurologique chronique qui touche près de 70 millions de personnes dans le monde [1, 2]. Près de 80% des malades sont rencontrés dans les pays en développement, avec des répercussions culturelles, économiques et sociales du fait des stigmas qui entourent son étiologie [1]. La maladie atteint 0,5 - 0,8% de la population, soit 1 sur 200 sujets [3]. Des taux élevés (2,2 à 58‰) sont rapportés dans certains foyers particuliers en Afrique [1]. Au Cameroun, l’épilepsie représente 10 à 16% des consultations en neurologie [4, 5]. Les étiologies des épilepsies sont très variées et résultent de la conjonction de facteurs génétiques, périnataux, d'anomalies du développement cortical, de lésions traumatiques crâniennes qui peuvent être cicatricielles, les maladies infectieuses, les tumeurs cérébrales, les accidents vasculaires cérébraux, les intoxications [6, 7]. L’épilepsie affecte le développement cognitif et constitue un frein au développement. Elle peut être à l'origine de handicap physique ou mental. Les données issues des formations sanitaires indiquent un nombre élevé de cas d’épilepsie dans le village de Bangoua. En 1995, une prévalence de 1,72% a été rapportée chez les patients consultant à l'hôpital de Bangoua, indiquant que cette localité est un foyer d’épilepsie [6]. Pour l'année 2012, les données hospitalières indiquent 147 nouveaux cas d’épilepsie sur 4044 patients consultés, dont une prévalence de 3,63%. Malgré les actions de sensibilisation faites par le personnel de santé, l’épilepsie reste pour la population locale une maladie mystérieuse, provoquée par le contact avec une plante, ou par la sorcellerie. La présente étude exploratoire a été conçue pour déterminer le niveau d'association entre les facteurs obstétricaux, infectieux et traumatiques et l’épilepsie à Bangoua.

## Méthodes

La présente étude cas-témoins a été réalisée dans la localité de Bangoua, département du Ndé, région de l'Ouest du Cameroun. Le village de Bangoua est situé à 12 km de la ville de Bangangté. Il a une superficie de 74 km^2^ et est composé de 18 quartiers. La population estimée à 8000 habitants est majoritairement de l'ethnie Bamiléké. La collecte des données a été faite dans les quartiers de Bangoua, impliquant des patients identifiés à partir des registres hospitaliers. Les patients épileptiques consentants (n = 70), ainsi que leurs témoins non épileptiques appariés selon l’âge et le sexe ont été recrutés du 4 août au 20 octobre 2008. Les témoins de même sexe que les cas, d’âge ± 5ans, étaient choisis dans la même famille que les malades. Le diagnostic de l’épilepsie était retenu lorsqu'un patient avait rapporté au moins deux crises d’épilepsie non provoquées au cours des deux dernières années. Les patients inclus étaient des épileptiques connus vivant dans la localité de Bangoua. L'outil principal de collecte des données était une adaptation du questionnaire d'investigation de l’épilepsie dans les pays tropicaux [8]. Il comportait 30 questions couvrant les items relatifs aux caractéristiques sociodémographiques (âge, sexe, statut marital, profession), les antécédents familiaux et obstétricaux, les antécédents infectieux (méningite, encéphalite, onchocercose), les antécédents traumatiques de la région céphalique, le contact avec les animaux domestiques tels chien, chat, mouton, chèvre, porc pour l'exposition à la cysticercose. Un examen physique a été mené pour rechercher des cicatrices au niveau de la tête et des nodules, dépigmentation, dermite onchocerquiennes. L'histoire de problèmes obstétricaux à la naissance susceptibles de provoquer une atteinte du système nerveux central a également été recueillie.

Le consentement des patients ou celui de leur représentant légal (pour les mineurs) était assuré avant le recrutement. Les participants étaient informés du fait que les informations collectées étaient anonymes. Le protocole de l’étude avait obtenu la clairance du comité national d’éthique avant le début du recrutement. Les données catégorielles et ordinales ont été exprimées en pourcentage (%) et les variables quantitatives en moyenne ± écart-type. Les données ont été saisies, contrôlées et analysées grâce au logiciel EPI-Info version 5.3.1. Le test T de Student a été utilisé pour comparer les moyennes dans les deux groupes. Pour les variables qualitatives, la comparaison s'est faite avec le rapport des côtes (RC) et un intervalle de confiance à 95%. Le model logistique en analyse des données groupées a été utilisé pour Calculer les rapports de côtes; les valeurs P y relatives ont utilisé le test de Fisher lorsque les observations attendues dans une cellule au moins étaient inférieures à 5 et le test de Chi-deux dans le cas contraire. Pour les variables ordinales, le test de tendance Cochran-Armitage test a été utilisé [9, 10]. Les valeurs-p inférieures à 5% étaient considérées statistiquement significatives.

## Résultats

Les patients épileptiques (n = 70) et les témoins non épileptiques (n = 70) étaient tous de l'ethnie Bamiléké. L’âge des patients variait de 6 ans à 65 ans avec une moyenne de 26,7 ± 10,6 ans. La moyenne d’âge des témoins était de 30,6 ± 14,9 ans avec des extrêmes de 6 et 70 ans. La différence d’âge entre les patients épileptiques et les témoins n’était pas statistiquement significative (p = 0,2) (Tableau 1). Le sexe masculin prédominait chez les patients épileptiques, représentant 54,3% contre 45,7% chez les témoins. La différence de sexe entre épileptiques et témoins n’était pas statistiquement significative (p = 0,3).

**Tableau 1:** Caractéristiques socio-démographiques des patients et témoins

Caractéristique	Patients		Témoins		RC	IC à 95%	P^++^
	Effectif	Pourcentage (%)	Effectif	Pourcentage (%)			
**Sexe**							
Masculin^+^	38	54,3	32	45,7	1		
Féminin	32	45,7	38	54,3	0,71	0,35-1,45	0,31
**Age (** *P pour le test de tendance de Cochran-Armitage = 0,70* **)**							
[0-15] ^+^	7	10,0	11	15,7	1		
[15-30]	37	52,9	32	45,7	1,82	0,56-5,97	0,27
30 et plus	26	37,1	27	38,6	1,51	0,45-5,17	0,45
**Statut matrimonial**							
Marié^+^	9	12,9	43	61,4	1		
Célibataire	61	87,1	27	38,6	10,79	4,31-27,79	0,000^***^
**Niveau d'instruction**							
Primaire et moins	48	68,6	43	61,4	1,37	0,64-2,92	0,34
Secondaire et plus^+^	22	31,4	27	38,6	1		
**Profession (** ***P du test de Fisher = 0.024*** **)**							
Emploi rémunéré^+^	16	22,9	26	37,1	1		
Agriculteur ou éleveur	41	58,6	25	35,7	2,67	1,12-6,41	0,015^*^
Elève ou étudiant	8	11,4	16	22,9	0,81	0,25-2,63	0,70
Sans emploi	5	7,1	3	4,3	2,71	0,47-16,99	0,20

**Antécédents familiaux:** plus de la moitié (57,1%) des patients épileptiques avaient des antécédents familiaux d’épilepsie. Parmi ceux-ci, 11,4% avaient deux des membres de leur famille qui étaient épileptiques et 10% avaient trois malades épileptiques ou plus (Tableau 2).

**Tableau 2:** Répartition des patients épileptiques par famille

Nombre de malades dans la famille	Effectif	Pourcentage(%)
Aucun	31	44,3
1	24	34,3
2	08	11,4
3 et +	07	10

**Antécédents obstétricaux:** les antécédents obstétricaux étaient inconnus pour 38,6% des participants épileptiques et 28,6% des non-épileptiques. Chez les patients épileptiques, l’évolution de la grossesse avait été anormale dans 42,9% des cas. Pour les témoins, 25,7% avaient rapporté une pathologie pendant la grossesse (p= 0,0024). Les pathologies dont avaient souffert les mères de patients épileptiques étaient le paludisme (n= 10), un état de mal épileptique (n= 3), une infection urinaire (n= 8) ou une éclampsie (n= 3). Les médicaments consommés pendant la grossesse étaient des antiépileptiques (n= 9), des antibiotiques (n= 12), des antipaludéens (n= 15) ou des potions d'infusions de plantes locales non identifiées par l’équipe (n= 6) (Tableau 3).

**Tableau 3:** Antécédents obstétricaux des patients et témoins

Caractéristique	Patients		Témoins		RC	IC à 95%	P++
	Effectif	Pourcentage (%)	Effectif	Pourcentage (%)			
**Accouchement à domicile**							
Oui	7	10,0	4	5,7	1,83	0,45-7,89	0,35
Non+	63	90,0	66	94,3	1		
**Césarienne**							
Oui	2	2,9	3	4,3	0,66	0,07-5,04	0,65
Non+	68	97,1	67	95,7	1		
**Prématurité**							
Oui	3	4,3	4	5,7	0,74	0,13-4,11	0,70
Non+	67	95,7	66	94,3	1		
**Réanimation à la naissance**							
Oui	4	5,7	8	11,4	0,47	0,11-1,84	0,23
Non+	66	94,3	62	88,6	1		
**Accouchement dystocique**							
Oui	2	2,9	2	2,9	1,00	0,10-10,29	1,00
Non+	68	97,1	68	97,1	1		
**Evolution de la grossesse (** ***P pour le test de Chi-deux de Pearson = 0,002*** **)**							
Ne sait pas	27	38,6	20	28,6	3,32	1,29-8,71	0,0057**
Anormale	30	42,8	18	25,7	4,10	1,58-10,82	0,001***
Normal+	13	18,6	32	45,7	1		

*** P ≤ 0,001

** P ≤ 0,01

+ Niveau de référence

IC = Intervalle de confiance

RC = Rapport des Côtes

++ Dans cette colonne, en dehors de l’évolution de la grossesse dont les Ps sont issus du Chi-deux, les autres sont du test de Fisher

**Antécédents infectieux:** les antécédents d'atteintes du système nerveux central étaient deux fois plus fréquents (p = 0,005) chez les participants épileptiques (38,6%) que chez les témoins (17,4%) (Tableau 4). De même, les signes d'onchocercose étaient six fois plus fréquents chez les participants épileptiques (12,9% contre 2,9%). Le contact avec les porcs était plus fréquent chez des patients épileptiques (33%) que chez les témoins (27%) mais la différence n’était pas significative.

**Tableau 4:** Antécédents infectieux des patients et des témoins

Caractéristique	Patients		Témoins		RC	IC à 95%	P++
	Effectif	Pourcentage (%)	Effectif	Pourcentage (%)			
**Méningite ou encéphalite**							
Oui	27	38,5	12	17,4	3,03	1,30-7,20	0,0047**
Non+	43	61,5	58	82,6	1		
**Onchocercose**							
Oui	9	12,9	2	2,9	5,02	1,13-34,87	0,032*
Non+	61	87,1	68	97,1	1		
**Contacts avec les porcs**							
Oui	19	27,1	23	32,9	0,76	0,35-1,67	0,46
Non+	51	72,9	47	67,1	1		

* P < 0.05

** P < 0.01

+ Niveau de référence

IC = Intervalle de confiance; RC = Rapport des Côtes

++ Dans cette colonne, le P pour ***Onchocercose*** est du test de Fisher, les deux autres sont du test du Chi-deux

**Figure 1:**
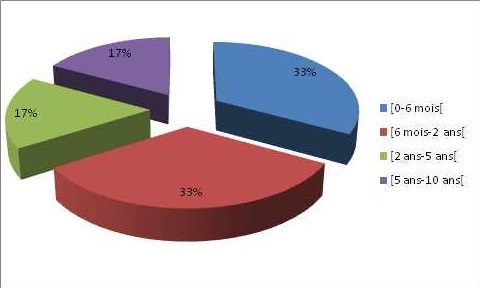
Délai d'apparition des signes d’épilepsie post traumatisme crânio-encéphalique

**Antécédents de traumatisme crânio-encéphalique:** les antécédents traumatiques crânio-encéphaliques étaient rapportés deux fois plus chez les participants épileptiques que chez les témoins (8,6% contre 4,3%). Pour 60% des sujets épileptiques, la première crise était survenue 6-24 mois après l'incident traumatique (Figure 1). La cause du traumatisme était une chute accidentelle (4,3%), un accident de la voie publique (2,9%), une bastonnade (1,4%). Les facteurs associés à l’épilepsie étaient la profession d'agriculteur ou d’éleveur et le statut de célibataire. Les potentiels facteurs de risque obstétricaux étaient le fait d’être né d'une grossesse d’évolution anormale. Les facteurs infectieux associés étaient un antécédent de méningite, d'encéphalite ou d'onchocercose.

## Discussion

L’âge moyen des patients épileptiques (26,7 ans) relevé dans la présente étude est plus faible que ceux rapportés au Mali (29,5 ans) [11]. Elle est cependant plus élevée que celui rapporté dans la vallée du Mbam au Cameroun (17,7 ± 8,4 ans) [12]. Cette différence pourrait être liée au processus de sélection des participants à ces études. La prédominance du sexe masculin relevée dans la présente étude corrobore les résultats rapportés en Europe et dans les pays en développement [3, 13]. Pendant l'adolescence et l’âge adulte, les hommes sont plus exposés aux traumatismes crânio-encéphaliques à travers les activités de cueillette de fruits, le roulage des engins dont ceux à deux roues (motos taxis) et lors des jeux [3, 13].

Le taux élevé de sous-scolarisation chez les patients épileptiques comparativement aux témoins corrobore celui trouvé dans la vallée du Mbam, où seuls 6,7% des patients atteignent l’école secondaire [14]. Les épilepsies symptomatiques s'accompagnent de lésions cérébrales et par conséquent un retard mental et intellectuel à l'origine de l'exclusion scolaire. Par ailleurs, les tabous qui entourent l’épilepsie en Afrique poussent les familles à garder les enfants à la maison et quelques uns sont exclus de l’école en raison des crises et la peur d'une contagiosité [15]. Le fait que plus de la moitié des patients épileptiques (57,1%) aient au moins un patient épileptique dans la famille est en faveur d'un probable déterminant génétique de l’épilepsie à Bangoua. Un antécédent familial d’épilepsie était retrouvé chez 7,7-63,4% des patients en milieu rural au Cameroun [16], au Sénégal [17], en Ethiopie [18], au Kenya [19], et en Tanzanie [20]. L'analyse des facteurs génétiques dans les principaux syndromes épileptiques pourrait permettre d’élargir les connaissances sur l’épileptogenèse. Certains auteurs distinguent les épilepsies à hérédité mendélienne autosomique dominante, mendélienne autosomique récessive et les épilepsies à hérédité complexe [21]. L'identification des gènes à l'origine de l’épilepsie aura des implications pour le diagnostic, ou pour prédire la survenue des crises [22]. Dans le cadre de la présente étude, nous avons inclut les oncles, tantes, cousins, neveux et nièces. Il nous est difficile de savoir si l'agrégation de cas d’épilepsie dans une même famille est liée à des facteurs génétiques ou à l'exposition à un facteur de l'environnement familial. Des études génétiques supplémentaires dans les familles les plus atteintes seront nécessaires.

Le taux élevé des facteurs prénataux prédisposant chez les patients épileptiques par rapport aux témoins évoquerait une contribution des facteurs prénataux dans la survenue de l’épilepsie. Ces résultats se rapprochent de ceux d'une étude antérieure où une fièvre maternelle était prédominante dans la population épileptique [16]. La fréquence des naissances à domicile plus élevée chez les patients épileptiques que chez les témoins corrobore les résultats obtenus au Congo [23]. La naissance à domicile accroit le risque d'accouchement septique, pouvant causer des infections néonatales qui pourraient contribuer à la survenue d'une atteinte neurologique dont une épilepsie. Une fréquence élevée de prématurité [23], le retard du cri à la naissance, les anomalies de la voix, les crises néonatales et les méningites en période néonatale ont été rapportés comme facteurs prédisposant à la survenue de l’épilepsie [23, 24]. Une hypoxie néonatale est responsable d'une épilepsie ultérieure et le risque de survenue de l’épilepsie post anoxo-ischémique est fonction de facteurs environnementaux, d'une prédisposition génétique et de l’âge de survenue de l'anoxie [25].

Les maladies infectieuses dont la méningite en période néonatale ont été incriminées dans la survenue de l’épilepsie au Mali [11]. Les infections du SNC peuvent provoquer une épilepsie séquellaire. Les infections parenchymateuses (encéphalite virale, méningo-encéphalite bactérienne, abcès cérébraux) sont particulièrement épileptogènes [23]. L'antécédent d'onchocercose retrouvé 6 fois plus chez les patients épileptiques corrobore les observations réalisées au Cameroun [14, 26]. L'importance de l'onchocercose dans la survenue de l’épilepsie a fait l'objet de plusieurs investigations aux résultats discordants [27, 28]. Une méta-analyse de neuf études Africaines montre un risque relatif de 1,21(CI 95% 0,99-1,47 (p = 0,06)) [28]. Cependant, une méta-analyse récente montre une association entre la maladie onchocerquienne et l’épilepsie. Ces auteurs indiquent qu'en moyenne, la prévalence de l’épilepsie est augmentée de 0,4% pour les 10% d'augmentation de la prévalence de l′onchocercose [29]. Le contact avec les porcs était présent chez 27,1% des patients épileptiques. Des observations non rapportées dans le présent travail font état de la quasi-absence de latrines et de la défécation humaine dans les porcheries. Ces pratiques courantes à Bangoua constituent des facteurs de risque associés à la transmission de Taenia solium, agent de la cysticercose porcine [30]. La neurocysticercose est connue comme déterminant de neuropathologies et donc de l’épilepsie [12, 27]. Une méta-analyse de 11 études Africaines montre un risque relatif de 3.4 à 3.8 (CI 95% 2.7-4.3; p < 0.001) de développer une épilepsie chez un patient souffrant de cysticercose [31].

Le taux élevé de traumatisme crânio-encéphalique chez les patients épileptiques par rapport aux témoins se rapproche des travaux du Laos [32]. Les traumatismes faisaient suite à une chute de hauteur et aux accidents domestiques [32]. Les épilepsies post traumatiques de la région crânienne peuvent être responsable de l’épilepsie par des mécanismes variés tels la contusion cérébrale, une fracture du crâne ou un hématome sous dural. La probabilité de survenue de l’épilepsie post traumatique augmente avec la gravité du traumatisme crânien et la présence de fracture du crâne au scanner [33]. Un monitoring continu par EEG est indispensable pour détecter les crises et/ou état de mal épileptique infra clinique après un traumatisme crânien, particulièrement chez les enfants de moins de 2 ans, ceux avec hémorragie intra-axiale au scanner et ceux avec agressions multiples [34].

## Conclusion

La présente étude a relevé que dans la localité de Bangoua, un antécédent familial d’épilepsie, le paludisme, l'onchocercose, une infection urinaire ou une éclampsie pendant la grossesse, un antécédent d'encéphalite sont associés à l’épilepsie. Les antécédents de traumatisme crânio-encéphalique, d'onchocercose, de contact avec les porcs sont associés à un risque élevé d’épilepsie.
